# Status of zinc nutrition in Bangladesh: the underlying associations

**DOI:** 10.1017/jns.2016.17

**Published:** 2016-06-06

**Authors:** Sabuktagin Rahman, Tahmeed Ahmed, Ahmed Shafiqur Rahman, Nurul Alam, A. M. Shamsir Ahmed, Santhia Ireen, Ireen Akhter Chowdhury, Fatima Parveen Chowdhury, S. M. Mustafizur Rahman

**Affiliations:** 1 Nutrition and Clinical Services Division, International Centre for Diarrhoeal Disease Research (ICDDR,B), 68 Shaheed Tajuddin Ahmed Sharani, Mohakhali Dhaka 1212, Bangladesh; 2 Health Systems and Population Studies Division, ICDDR,B, 68 Shaheed Tajuddin Ahmed Sharani, Mohakhali Dhaka 1212, Bangladesh; 3 School of Public Health, Faculty of Medicine and Biomedical Sciences, The University of Queensland, Herston, QLD, Australia; 4 UNICEF, Bangladesh; 5 Ayesha Memorial Medical College, Dhaka, Bangladesh; 6 Institute of Public Health Nutrition, Dhaka, Bangladesh; 7 Micronutrient Initiative, Dhaka, Bangladesh

**Keywords:** Zinc deficiency, Zinc intake, Children, Women in Bangladesh, Rural, urban and slum, AGP, α-1-acid glycoprotein, CRP, C-reactive protein, NPNLW, non-pregnant non-lactating women, PSAC, pre-school-age children, QC, quality control, SES, socio-economic status

## Abstract

Bangladesh is a country with a high burden of micronutrient malnutrition. Stunting affects 41 % of children aged under 5 years. Zn is one of the key micronutrients that is associated with stunting. The present study, as part of the national micronutrient survey 2011–2012, revealed for the first time the nationally representative prevalence of Zn deficiency and determined the associations of the condition. A cross-sectional ‘nationwide’ survey was conducted in pre-school-age children (6–59 months; PSAC) and non-pregnant non-lactating women (15–49 years; NPNLW). Multistage random sampling was done in 150 clusters; fifty in each of the rural, urban and slum strata. Data were analysed on 662 PSAC and 1073 NPNLW. Serum Zn was assayed by atomic absorption spectrophotometry. Zn deficiency was defined as serum Zn of <9·9 and <10·1 µmol/l in PSAC and NPNLW, respectively. The national prevalence of Zn deficiency was 44·6 and 57·3 % in PSAC and NPNLW, respectively. In PSAC, it was 29·5, 48·6 and 51·7 %, respectively, in urban, rural and slum strata. Household expenses (β = 0·13; *P* = 0·007), Hb (β = 0·10; *P* = 0·005), intake of animal-source Zn (β = 0·096; *P* = 0·02) and asset score (β = 0·11; *P* = 0·03) were positively associated with serum Zn in NPNLW. Residence in an urban area (β = 0·33; *P* = 0·03) and intake of plant-origin Zn (β = −0·13; *P* = 0·038) determined higher and lower status of Zn in PSAC, respectively. Zn deficiency was highly prevalent in Bangladesh, and it was principally related to inadequate quality of diet. To improve Zn nutrition, Bangladesh needs to strengthen research and programmes related to Zn biofortification, fortification and phytate-reducing technologies in the food system in the short and medium term. In addition, promotion of animal-source Zn for all is important in the long run.

Zn is one of the most important trace elements involved in human metabolism. It is implicated in all major biochemical metabolism and plays multiple roles in the maintenance of genetic material, including transcription of DNA, translation of RNA and cellular division^(^^1^^)^. Zn supplementation produces highly significant positive responses in linear growth and weight gain^(^^2^^)^. On the other hand, Zn deficiency is associated with stunted linear growth and diminished immune function. Supplementation of the mineral is also associated with lessening of diarrheal duration and respiratory tract infection in children^(^^2^^)^. Childhood stunting in children aged under 5 years is widely recognised as a proxy indicator of Zn deficiency. However, not until the present study was conducted in 2011–2012 were nationwide data of Zn deficiency available in Bangladesh, a country with a high prevalence of stunting in children aged under 5 years (41 %)^(^^3^^)^. The present study has revealed for the first time in Bangladesh the national estimates of subclinical Zn deficiency and the underlying associations of the condition.

## Methods

### Sampling and study population

The present study is a part of the national micronutrient survey 2011–2012 of Bangladesh. A multi-stage random sampling procedure was applied to select the study participants from the rural, urban and the slum domains distributed all over the country. In the first stage, 150 clusters (fifty clusters in each domain) from the 15 000 clusters of the Bangladesh Multiple Indicator Cluster Sampling (MICS)^(^^4^^)^ frame were randomly selected. In the second stage a segment of fifty households was randomly chosen from each of the selected clusters and in the final stage, twenty households were selected randomly from the fifty-household segment; these twenty households formed the definitive sampling frame from which the required number of study participants was selected.

The sample size (Supplementary Table S3) for assessing Zn deficiency was representative of the stratum; however, combining all the three strata together and with application of population weight, nationally representative estimates were obtained. Data were collected in two population groups – pre-school-age children (PSAC; 6–59 months) and non-pregnant non-lactating women (NPNLW; 15–49 years).

### Data and blood sample collection

Data on socio-economic status (SES), household possession of assets, household construction material, household food insecurity, morbidity of children and food intake of the study participants were analysed. SES was assessed by constructing the wealth index^(^^5^^)^. Household food insecurity was assessed by the Household Food Insecurity Access Scale (HFIAS) for measurement of food access^(^^6^^)^. In order to assess food consumption, a semi-quantitative FFQ was used taking into consideration commonly consumed Bangladeshi foods^(^^7^^–^^10^^)^. The semi-quantitative FFQ assessed consumption over the preceding 7 d of the survey. The tool was administered by the field data collectors. For NPNLW, the study respondent provided the response; however, for PSAC, the mother or primary caretaker responded. The tools did not require the respondents to have any reading or writing skills necessary. The respondent was asked to tell about the number of portions (servings) of particular foods that she and/or her child had consumed over the past 7 d. She was required to indicate the portion size (g, ml) of the consumption from the standardised food photographs or commonly used household utensils, which were displayed and explained at the time of the interview. The number of portions consumed over the past 7 d multiplied by the average size of the portions yielded the total weekly consumption (g, ml). Raw food weight was calculated by using appropriate conversion factors^(^^11^^)^. The nutrient value for Zn was calculated per 100 g of raw food consumed using an updated Food Composition Table on Bangladeshi food^(^^12^^)^. The prevalence of Zn intake inadequacy was measured by the estimated average requirement (EAR) cut-point method^(^^1^^)^. For children, we defined dietary Zn inadequacy using the EAR for 2- to 3-year-old children (<2 mg/d)^(^^1^^)^. For women, dietary Zn inadequacy was calculated by using lower bioavailability with an unrefined, cereal-based diet (<7 mg/d)^(^^1^^)^. Phytate is a chemical substance present in plant foods which acts as a chelator of minerals, including Zn. The inhibitory effect of phytate on Zn absorption appears to follow a dose-dependent response, and the phytate:Zn molar ratio of the diet has been used to estimate the proportion of absorbable Zn^(^^1^^)^. To classify populations according to mixed or cereal-based diet types, the phytate:Zn molar ratio of foods or diets was calculated according to the methods described in the technical document of the International Zinc Nutrition Consultative Group^(^^1^^)^. Blood samples were collected in a centralised location, such as school, health centre, non-governmental organisation (NGO) office, developed as temporary sample collection centres. The selected survey respondents were given a token number indicating their names and identification and guided to appear at the collection centres. Venous blood was collected in trace element-free venoject tubes. The blood tubes were placed in a cool box and allowed to clot. The whole blood was centrifuged (Portable Centrifuge, 3000–3500 rpm) at the field collection centre and the serum was aliquoted in cryovials (trace element free) using a disposable pipette. The cryovials were stored in a −20°C freezer. Aliquoted serum samples were transferred in dry ice to the Nutritional Biochemistry Laboratory at the International Centre for Diarrhoeal Disease Research, Bangladesh (ICDDR,B), and stored in a −70°C freezer. The study received ethical approval from the Institutional Review Board of ICDDR,B. Written informed consent was taken from all study participants.

### Biochemical analysis

We assayed serum Zn by atomic absorption spectrophotometry (Shimadzu AA-7000). C-reactive protein (CRP) and α-1-acid glycoprotein (AGP) were analysed by sandwich ELISA (Dynex Technologies Inc.). Hb was assessed using HemoCue Hb 301 (Hemocue AB) on venous blood.

### Adjusting for infection

Serum Zn level was adjusted for infection by estimating biomarkers of infection – CRP and AGP. We performed the adjustment for elevated levels of CRP (>10·0 mg/l) and AGP (>1·0 g/l) by deriving the correction factors following the methods described by Thurnham *et al.*^(^^13^^)^ and Engle-Stone *et al.*^(^^14^^)^.

### Quality control

All biochemical analyses were carried out in the nutritional biochemistry laboratory of ICDDR,B. To control the quality of the laboratory analyses pooled serum and commercial quality-control (QC) material (Bi-level serum toxicology control; UTAK Laboratories, Inc.) were used for Zn. Pooled serum had the assigned value against the standard reference material. The pooled serum was stored in a freezer (−20°C) and analysed with every batch of samples along with the commercial control material. If the values of these preliminary analyses had fallen within the assigned ranges for the QC, the regular QC assays (pool and commercial) for all samples were performed. The mean values for the pool and the commercial QC along with standard deviations and 95 % CI were calculated. For Zn, the CV against the pooled serum, QC-normal and QC-high were 4·5, 4·1 and 4·0 %, respectively. Precinorm protein and Precipath protein (Roche Diagnostics GmbH), as QC, were used to check the accuracy and precision of CRP and AGP. The CV for CRP and AGP measurement were 3·9 and 5·9 %, respectively. We participated in the VITAL-EQA (Centers for Diseases Control) for CRP.

### Sample size

The sample size to estimate Zn undernutrition was calculated based on the prevalence of low serum Zn observed in a contemporary study in Bangladesh^(^^15^^)^. The required sample size for PSAC and NPNLW was 969 and 1500, respectively, taking into consideration an attrition factor of 1·2 and a design effect 2·0. However, the final analysed sample number was 662 and 1073, respectively, taking into consideration the availability of both the biomarkers of infection (CRP and AGP) for analysis, in order to adjust for infection. In addition, losses in sample number occurred due to inadequate serum in aliquots, haemolysis, and error in labelling of sample identification by the field staff. This resulted in some overall loss of precision in the prevalence estimates: 10·3 % precision instead of the stipulated 7 % in PSAC and 6·2 % precision in NPNLW in place of 6 % (Table 1 and Supplementary Table S3).

**Table 1.** Prevalence of zinc deficiency*†‡ (Number of subjects, percentages and 95 % confidence intervals)

Population	*n*	%	95% CI
PSAC§			
National	662	44·6	34·3, 54·9
Rural	228	48·6	35·8, 61·4
Urban	236	29·5	17·7, 41·3
Slum	198	51·7	40·8, 62·7
NPNLW∥			
National	1073	57·3	51·1, 63·4
Rural	391	57·5	49·9, 65·1
Urban	359	54·5	45·5, 63·6
Slum	323	66·4	55·1, 77·6

PSAC, pre-school-age children; NPNLW, non-pregnant non-lactating women; CRP, C-reactive protein; AGP, α-1-acid glycoprotein.

* Zn deficiency is defined as serum Zn level of <9·9 µmol/l in PSAC and <10·1 µmol/l in NPNLW^(^^1^^)^.

† Adjusted for elevated CRP (>10 mg/l) or elevated AGP (>1 g/l) by mathematical correction^(^^12^^,^^13^^)^.

‡ Estimates weighted to represent at the population level.

§ Age 6–59 months.

∥ Age 15–49 years.

### Statistical analysis

We performed the statistical analyses using STATA 10.0 SE (StataCorp LP). We calculated the key proportion estimates with a 95 % CI; the mean estimates were calculated with standard deviations through one-way ANOVA. Pearson's χ^2^ test was used to compare proportions. Projected estimates of the population in the three strata (rural, urban and slum) in 2011 were 122·6, 23·3 and 5·5 million, respectively^(^^16^^,^^17^^)^. A total of fifty primary sampling units (PSU) were selected from each stratum, hence resulting in differential selection probabilities and representation across the strata. Sampling weights were applied to the households in each stratum to compensate for the differential representation and to derive weighted national estimates combining the estimates of the three strata. Prior to performing statistical tests, we treated the variables with skewed distribution with logarithmic transformation to convert into normal distribution. Statistical analyses, e.g. proportion estimates, one-way ANOVA and multivariate regression analyses were performed on weighted data. Through using the cluster option in STATA, the analyses, including the regression models, were adjusted for clustering of data at the unit of the randomisation level (PSU). The relevant socio-economic, demographic and dietary intake predictor variables which are logically presumed to have association with serum Zn were entered in univariate linear regression or bivariate analysis. If there was a significant association with serum Zn at *P* < 0·05 and it had the expected sign mark (i.e. plus or minus sign of the coefficient according to the logical expectation), the variables were selected for the multivariate regression^(^^18^^)^. Interaction analyses were performed taking into consideration the variables that logically appear to be interacting in determining serum Zn status. The predictor variables, which are assumed to have interaction, were entered in the initial regression analyses as an interaction term along with the interacting predictors. If the regression coefficient for the interaction term was non-significant (*P* ≥ 0·05), it was omitted; however, in cases where the interaction term was statistically significant (*P* < 0·05), it was retained and entered into the final multivariate model^(^^19^^)^.

Cronbach's α reliability coefficients for internal consistency of data were calculated considering the variables of SES, dietary consumption, household food insecurity and serum level of Zn. Cronbach's α in data was 0·8179 and 0·8159, respectively, in PSAC and NPNLW, which is suggestive of good internal consistency^(^^20^^)^.

## Results

The national prevalence of Zn deficiency in pre-school children was 44·6 % (Table 1). It was 29·5, 48·6 and 51·7 %, respectively, in the urban, rural and slum strata. Of the NPNLW, 57·3 % suffered from Zn deficiency at the national level; the prevalence being highest in women living in slums (66·4 %). The national mean of serum Zn was 10·2 and 10·04 µmol/l in PSAC and the NPNLW, respectively. The mean level of serum Zn appeared to be higher in the urban stratum than in the slum stratum in both the populations (*P* < 0·001, *P* = 0·017, respectively) (Table 2).

**Table 2.** Mean zinc concentration in serum (μmol/l)‡§ (Mean values and standard deviations)

	PSAC∥		NPNLW¶	
	Mean‡‡	sd	Mean‡‡	sd
National	10·25	2·34	10·04	1·72
Rural	10·04	1·75	10·06**	1·76
Urban	11·02***†††	3·68	10·05*	1·55
Slum	9·89	1·60	9·67	1·67

PSAC, pre-school-age children; NPNLW, non-pregnant non-lactating women; CRP, C-reactive protein; AGP, α-1-acid glycoprotein.

Mean value was significantly different from that for slum: *P = 0·017, ** *P* = 0·012, *** *P* < 0·001.

††† Mean value was significantly different from that for rural (*P* < 0·001).

‡ Adjusted for elevated CRP (>10 mg/l) or elevated AGP (>1 g/l) by mathematical correction^(^^11^^,^^12^^)^.

§ Estimates weighted to represent at the population level.

∥ Age 6–59 months.

¶ Age 15–49 years.

‡‡ One-way ANOVA.

**Fig. 1.**
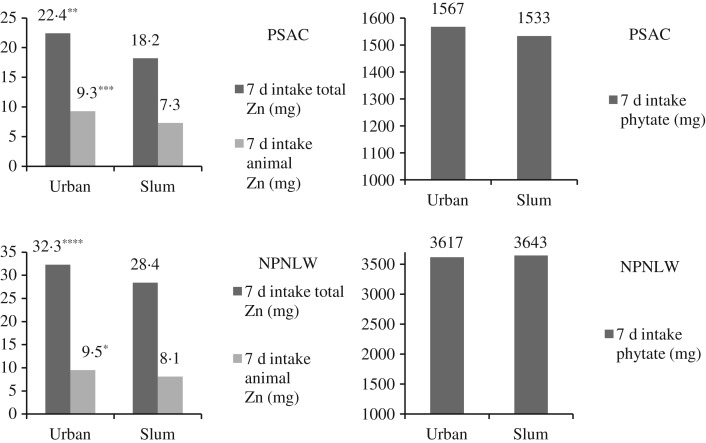
Comparative intake of dietary zinc and phytate in urban and slum strata. Intakes of total zinc and animal-source zinc were significantly higher in the urban stratum than in the slum stratum in both the pre-school-age children (PSAC) and non-pregnant non-lactating women (NPNLW) populations: 22·4 mg/7 d (urban) *v.* 18·2 mg/7 d (slum) (*P* = 0·004) and 9·3 mg/7 d (urban) *v.* 7·3 mg/7 d (slum) (*P* = 0·005), respectively, for total and animal-origin zinc in PSAC. However, intake of phytate was at similar levels: 1567 mg/7 d (urban) *v.* 1533 mg/7 d (slum) (NS) in PSAC. Similar profiles of the intake were observed in NPNLW. Significantly different from slum: * *P* = 0·01, ** *P* = 0·004, *** *P* = 0·005, **** *P* = 0·001.

The prevalence of inadequate intake of Zn in PSAC was 32·6 % at the national level. The respective estimates for rural, urban and slum domains were 33·0, 28·7 and 44·1 %. In NPNLW, 91·1 % had inadequate intake of Zn at the national level; by and large the estimates remained at similar levels across the strata (Table 3). The national average consumption of dietary Zn in PSAC was 3·1 mg/d, the urban intake being higher than in slums (3·2 *v*. 2·6 mg/d; *P* = 0·004). At the national level mean intake of Zn in NPNLW was 4·2 mg/d. The urban women consumed higher amounts than their peers from slums (4·6 mg *v*. 4·1 mg/d; *P* = 0·001; Table 3). Fig. 1 further depicts the relative intake profile.

**Table 3.** Dietary intake of zinc and prevalence of inadequacy of zinc intake (Mean values and standard deviations; percentages with their standard errors)

	Intake of Zn (mg/d)†								Prevalence of inadequate Zn intake (%)‡							
	National		Rural		Urban		Slum		National		Rural		Urban		Slum	
	Mean	sd	Mean	sd	Mean	sd	Mean	sd	%	se	%	se	%	se	%	se
PSAC	3·1	2·1	3·1	2·1	3·2*	2·0	2·6	1·5	32·6	5·0	33·0	6·5	28·7	5·5	44·1	4·6
NPNLW	4·2	2·5	4·1	2·2	4·6**	2·3	4·1	2·0	91·1	1·5	91·5	1·8	89·2	2·7	91·1	1·7

PSAC, pre-school-age children; NPNLW, non-pregnant non-lactating women.

Mean value was significantly different from that for slum: * *P* = 0·004, ** *P* = 0·001.

† One-way ANOVA.

‡ Inadequate Zn intake: <2 mg/d in PSAC; <7 mg/d in NPNLW^(^^1^^)^.

Less than 1·0 and 6·4 % of the NPNLW aged 15–18 and 19–49 years, respectively, met the RDA for Zn^(^^21^^)^. In PSAC, 44·7 % of the 2- to 3-year-olds and 11·4 % of the 4- to 5-year-olds met the RDA^(^^21^^)^ (Table 4). Mean intake of Zn was 2·8 and 3·2 mg/d in 2- to 3- and 4- to 5-year-old groups, respectively; it was 4·0 and 4·3 mg/d in NPNLW aged 15–18 and 19–49 years, respectively. Intake of animal-source Zn was 1·13–1·15 mg/d in PSAC and 1·28–1·33 mg/d in NPNLW according to age subgroups (Table 4). Intake of animal-origin Zn progressively increased as household SES and household expenses were higher; an increasing level of food insecurity was associated with progressively decreasing intake in children and women. However, intake of phytate remained at similar levels irrespective of SES and household food security status (Table 5).

**Table 4.** Intake of zinc *v*. RDA (Mean values with their standard errors; percentages with their standard errors)

		B: % population meeting RDA		C: intake (mg/d)			E: intake of animal Zn (mg/d)		
Population	A: RDA (mg/d)*	%	se	Mean	se	D: intake as % of RDA†	Mean	se	F: % animal Zn to total intake‡
PSAC 2–3 years	3	44·7	5·3	2·8	0·2	93·6	1·13	0·1	40·2
PSAC 4–5 years	5	11·4	3·4	3·2	0·3	64·0	1·15	0·1	35·9
NPNLW 15–18 years	9	0·5	0·03	4·0	0·2	44·4	1·33	0·1	33·2
NPNLW 19–49 years	8	6·4	1·3	4·3	0·2	53·6	1·28	0·1	29·8

PSAC, pre-school-age children; NPNLW, non-pregnant non-lactating women.

* Institute of Medicine^(^^21^^)^.

† D = C/A × 100.

‡ F = E/C × 100.

**Table 5.** Intake of animal-source zinc and phytate by socio-economic status (SES) and household food insecurity (Mean values and standard deviations)

	Intake of animal-source Zn (mg/d)		7-d intake of phytate (mg)		Household spending (USD/month)	
	Mean‡	sd	Mean‡	sd	Mean‡	sd
NPNLW						
Poorest	0·8^a^	0·7	3388	1693	76·8^a^	30·4
Middle	1·4^b^	1·1	3533	1367	125·5^b^	57·1
Richest	2·1^c^	1·2	3521	1335	225·9^c^	121·3
PSAC						
Poorest	0·6^a^	0·7	1636	698	76·3^a^	28·9
Middle	1·3^b^	0·8	1782	712	124·3^b^	72·1
Richest	1·8^c^	1·1	1546	659	212·5^c^	111·1
NPNLW						
FS	1·6	1·1	3524	1364	146·1	96·9
MI	0·9*	0·8	3626	1806	101·9*	48·9
SI	0·7*	0·7	3281	1634	98·7*	58·5
PSAC						
FS	1·5	1·0	1700	574	144·4	92·1
MI	0·9*†	0·9	1756	1126	95·2*	41·9
SI	0·5*	0·5	1562	765	81·1*	49·8

USD, US dollars; NPNLW, non-pregnant non-lactating women; PSAC, pre-school-age children; FS, food secure; MI, moderate insecure; SI, severe insecure.

^a,b,c^ Mean values within a column within a category with unlike letters were significantly different (*P* < 0·001).

* Mean value was significantly different from that for FS (*P* < 0·001).

† Mean value was significantly different from that for SI (*P* = 0·001).

‡ One-way ANOVA.

According to dietary patterns of ‘mixed’ and ‘cereal-based’ diets, inadequacy of Zn intake in women was pervasively prevalent at 90 and 100 %, respectively. As per dietary type mean intakes of Zn were 4·5 and 2·4 mg/d, respectively (*P* < 0·001; Table 6). In accordance with the absorption pattern for mixed and cereal-based diets^(^^1^^)^, the amount of absorbed Zn was 1·41 and 0·56 mg/d which fulfilled 75·8 and 30·1 % of physiological requirements, respectively. Intake of animal-source food, according to mixed and cereal-based diets was: meat, 27·6 *v*. 6·7 g/d (*P* < 0·001); fish, 58·6 *v*. 22·5 g/d (*P* < 0·001); and eggs, 11·7 *v*. 3·4 g/d (*P* < 0·001), respectively. Intake of cereals was 337·1 and 457·4 g/d, respectively (*P* < 0·001).

**Table 6.** Zinc status in non-pregnant non-lactating women sorted by dietary type and food intake (Percentages with their standard errors, and mean values and standard deviations)

	A: Zn deficiency		B: inadequate Zn intake†		C: total Zn intake (mg/d)					G: meat intake (g/d)		H: fish intake (g/d)		I: egg intake (g/d)		J: cereal intake (g/d)	
	%	se	%	se	Mean	sd	D: absorbable Zn intake (mg/d)‡§	E: physiological requirement (mg/d)∥	F: % of physiological requirement met¶	Mean	sd	Mean	sd	Mean	sd	Mean	sd
Mixed diet††	59·2*	3·3	90·1**	1·6	4·5**	2·2	1·41	1·86	75·8	27·6**	35·6	58·6**	44·3	11·7**	17·4	337·1**	132
Cereal-based diet‡‡	48·3	6·4	100·0	0	2·4	1·0	0·56	1·86	30·1	6·7	10·6	22·5	17·9	3·4	9·4	457·4	211

Significantly different from cereal-based diet: * *P* = 0·32, ** *P* < 0·001.

† Dietary Zn intake <7 mg/d (estimated average requirement) in women^(^^1^^)^.

‡ Absorption in women of 31 and 23 % for mixed and unrefined cereal-based diets, respectively^(^^1^^)^.

§ D = C × 0·31 and D = C × 0·23 for mixed and cereal based diets, respectively^(^^1^^)^.

∥ Physiological requirement of absorbed Zn in adult women^(^^1^^)^.

¶ F = D/E × 100.

†† Phytate-Zn molar ratio (4–18)^(^^1^^)^.

‡‡ Phytate-Zn molar ratio (>18)^(^^1^^)^.

By and large, there was an upward gradient in serum levels of Zn concurring with progressively increased intake of dietary Zn in PSAC; however, in NPNLW that trend was not observed (Table 7).

**Table 7.** Dietary intake of zinc and serum levels of zinc (Mean values with their standard errors)

	Intake of Zn (mg/d)		Serum Zn (μmol/l)	
Intake levels of Zn (mg/d)	Mean*	se	Mean*	se
PSAC				
2·5–3·49	3·0	0·04	9·74	0·24
3·5–4·49	3·9	0·03	9·81	0·26
4·5–5·49	4·9	0·05	10·18	0·50
5·5–6·49	6·1	0·07	10·42	0·66
NPNLW				
2·5–3·49	2·9	0·03	10·16	0·14
3·5–4·49	4·0	0·03	9·74	0·23
4·5–5·49	5·0	0·02	9·66	0·27
5·5–6·49	6·0	0·04	10·29	0·46
6·5–7·49	7·0	0·04	10·77	0·38
7·5–8·49	7·9	0·06	10·19	0·58

PSAC, pre-school-age children; NPNLW, non-pregnant non-lactating women.

* One-way ANOVA.

Table 8 shows the results of the multivariate linear regression analyses stating associations of serum Zn status in the studied populations after controlling for the relevant covariates. Multivariate linear regression analyses suggested that household expenses (β = 0·13; *P* = 0·007), Hb (β = 0·10; *P* = 0·005), intake of animal-source Zn (β = 0·096; *P* = 0·02) and asset score (β = 0·11; *P* = 0·03) determined higher levels of serum Zn in NPNLW. Non-agricultural occupation of the household head was nearly positively associated with serum Zn in NPNLW (β = 0·07; *P* = 0·054). Intake of plant-source Zn determined the lower status of Zn in NPNLW (β = −0·13; *P* = 0·001). Residence in an urban area was associated with higher serum Zn in PSAC compared with their peers from slum areas (β = 0·33; *P* = 0·03). Intake of plant-origin Zn was associated with lower status of Zn in PSAC (β = −0·13; *P* = 0·038), while intake of animal-source Zn was nearly associated with higher levels of serum Zn (β = 0·13; *P* = 0·07).

**Table 8.** Multivariate regression determining zinc level in serum in pre-school-age children (PSAC) and non-pregnant non-lactating women (NPNLW)

	PSAC*			NPNLW†		
Covariates	Coefficient	*P*	β	Coefficient	*P*	β
Hb	0·001	0·98	0·0009	0·18	0·005	0·10
Household expenses	0·18	0·43	0·06	0·42	0·007	0·13
Age	0·014	0·22	0·08	−0·012	−0·07	−0·07
Occupation of household head						
Non-agricultural (reference agricultural)	0·29	0·21	0·085	0·27	0·054	0·07
Women's education						
Secondary (reference below secondary)	0·17	0·59	0·033	−0·003	0·98	−0·0005
Intake of animal-source Zn	0·00003	0·07	0·13	0·00002	0·02	0·096
Intake of plant-source Zn	−0·000025	0·038	−0·13	−0·00002	0·001	−0·13
Asset score	0·08	0·58	0·05	0·23	0·03	0·11
Area of residence						
Urban (reference slum)	1·37	0·03	0·33	0·18	0·64	0·04
HFIAS score	0·04	0·12	0·11	0·02	0·10	0·066

HFIAS, Household Food Insecurity Access Scale.

* Interaction terms – (occupation of household head × intake of animal-source Zn), (occupation of household head × intake of plant-source Zn), (asset score × intake of animal-source Zn), (household expenses × intake of animal-source Zn), (stratum × intake of animal-source Zn), and (household expenses × educational status of women) were separately assessed through initial regression equations, along with component predictors. However, none of the interaction terms reached statistical significance level at *P* < 0·05 except for (household expenses × occupation of household head) (*P* = 0·049), which was considered in the final multivariate model; however, it had a non-significant interaction (*P* = 0·44) and as such was removed from the model.

† Interaction terms – all the interaction terms assessed for the PSAC were tested in NPNLW. In the initial regression analyses none had significant interaction except for the household expenses × intake of animal-source Zn (*P* = 0·03). It was considered in the final multivariate model where it is reported to have significant interaction (*P* = 0·01).

## Discussion

The finding of 44 % prevalence of subclinical Zn deficiency in preschool children was close to the contemporary stunting prevalence of 41 % in Bangladesh^(^^3^^)^, reaffirming the notion that the stunting estimate can be a proxy indicator of Zn undernutrition in children. The dietary intake of Zn in the present study (Table 3) was consistent with a contemporary study which reported intake of 2·5 mg in young children and 5·5 mg in women in two subdistricts^(^^15^^)^. The same study reported the prevalence of inadequate intake of Zn to be 22 and 94 % in children and women, respectively, which approximately corresponds to our findings (Table 3).

In PSAC, increasing intake of Zn to roughly twice the amount (from 3·0 to 6·1 mg) was associated with a 7 % increment in serum Zn level (from 9·74 to 10·42 µmol/l; Table 7). A recent meta-analysis pooling all eligible randomised controlled trials of Zn supplementation modelled that doubling of Zn intake was associated with a 9 % increment in serum Zn levels^(^^22^^)^. Hence our observation of a 7 % increase from this cross-sectional national survey is by and large consistent with the experimentally designed global model predicting Zn supplementation and its reflection in serum status. This is an important finding in relation to the designing of Zn promoting interventions for children in the country. In NPNLW, the approximated doubling of intake (from 2·9 to 6·0 mg, from 4·0 to 7·9 mg; Table 7) was associated with a less marked increase in serum Zn (1·2 and 4·6 %, respectively). Somewhat inconsistent Zn status in NPNLW in relation to dietary intake perhaps has resulted from the fact that Zn status varies with exercise, stress, starvation and timing of food intake^(^^23^^)^. Bangladeshi women are likely to experience these in day-to-day affairs. However, these factors were not explored in the survey.

The multivariate regression showed intake of animal-source Zn was associated with a higher level of serum Zn in NPNLW (Table 8). The underlying mechanism is unclear; however, other studies reported animal protein being associated with a greater percentage absorption of dietary Zn^(^^24^^,^^25^^)^.

Household expenses which signify household spending capacity were associated with a higher level of serum Zn in NPNLW. The underlying mechanism into this is not clear; however, it might be attributed to the higher consumption of higher bioavailable animal-origin Zn by women from households with higher financial status (Supplementary Table S1). Multivariate regression showed that Hb level was positively associated with serum Zn in NPNLW (Table 8). Complementing this, the prevalence of subclinical Zn deficiency was found to be higher in anaemic women than in non-anaemics (Supplementary Table S1). This might be explained by common dietary sources of Zn and Fe or the possible role of Zn in erythropoiesis^(^^26^^–^^28^^)^.

Multiple regressions showed that the PSAC living in urban areas had higher levels of serum Zn compared with slum areas. It is in agreement with a lower prevalence of subclinical Zn deficiency in urban PSAC than their peers in the slums (Table 1). The underlying reason is difficult to explain; however, we assume that a 23·1 and 27·4 % higher intake of total and animal-source Zn, respectively, might have accounted for the higher status of serum Zn in urban PSAC (Fig. 1). On the other hand, in NPNLW, urban residence was not associated with higher status of Zn (Table 8). Underlying this is perhaps the intakes of total and animal-source Zn which were only marginally higher (13·7 and 17·2 %, respectively; Fig. 1) in urban women, while the intake of phytate was at similar levels.

Household asset score determined higher status of Zn in NPNLW. This could be plausibly explained by differential intakes of higher bioavailable animal-origin Zn which was about 2·5 times higher in the ‘richest’ households than their peers from the ‘poorest’ (Table 5). However, in PSAC the asset score was not a predictor of Zn status. The reason for the differential relationship can be explained by the bioavailability of Zn, an issue that perhaps affects women more in Bangladesh. This is evident from the data in Table 5; for example, in the ‘poorest’ SES, while women's consumption of animal-source Zn was 33 % higher than in the ‘poorest’ PSAC (0·8 *v*. 0·6 mg/d), the intake of phytate (Zn chelator) was 207 % higher in the poor women, compared with the poor young children (3388 *v*. 1636 mg/7 d). Thus, the PSAC are likely to be affected less than the women in relation to bioavailability of Zn as a result of a lower consumption of phytate. This might lead to a relatively favourable bioavailability of Zn in PSAC across a wide range of SES.

Dietary intake of phytate was at similar levels across the SES or household food security status in both the populations (Table 5). This is perhaps one of the fundamental issues behind the population-wide high prevalence of subclinical Zn undernutrition in Bangladesh.

The strength of the study lies at the consideration of multiple domains – rural, urban and slum, where status of Zn nutrition and attribution of underlying correlates, such as, SES and food consumption status were different, and thus providing a representative status of Zn nutrition of the country. Although the semi-quantitative FFQ has been increasingly used and validated in other countries^(^^7^^–^^10^^)^, it is new in Bangladesh, and hence, it was not validated in Bangladeshi populations. While this is a limitation of the study, when comparing the intake data in the survey with another contemporary study in the country^(^^15^^)^ in nearly comparable population groups, the results appear to be similar (Table 3, Supplementary Table S2). Although the semi-quantitative FFQ has been validated for Zn^(^^7^^,^^8^^)^; it is not directly validated for phytate, which is a limitation of the study. However, the tool is validated for cereals, beans and dietary fibre^(^^7^^,^^8^^)^, and these food components are the major sources of phytate.

The timing of blood sample collection in relation to the last meal is important for assessing serum Zn status^(^^29^^)^. However, we could not arrange a time protocol for sample collection due to the limited amount of time to complete data collection in the clusters. Also, because the respondents visited the collection centres according to their convenience in the day, this was a limitation as well.

In conclusion, subclinical Zn deficiency is pervasively prevalent in Bangladesh in vulnerable populations. The worst hit by the condition are women, people living in lower SES and in the slums. A high burden of Zn undernutrition is related to the intake lower than the requirements, especially that of the higher bioavailable animal-source Zn and the high intake of plant-origin phytate-bound Zn. Bangladesh needs to strengthen research and programmes related to Zn supplementation, biofortification, industrial fortification and phytate-reducing technologies in the food system in the short and medium term, along with promotion of animal-source Zn for all in the long run.

## Supplementary material

For supplementary material accompanying this paper visit http://dx.doi.org/10.1017/jns.2016.17. click here to view supplementary material
