# Supplementary material for: Status of zinc nutrition in Bangladesh: the underlying associations
Source: J Nutr Sci. 2016 Jun 6;5:e25. doi: 10.1017/jns.2016.17 (PMC4976114; doi:10.1017/jns.2016.17)
Supplement: Supplementary file 1 [file S2048679016000173sup001.docx]

Supplementary Table 1: Underlying analyses into the multivariate regression findings for serum zinc

| Regression finding: Household expense was associated with higher level of serum zinc in NPNLW | | | | | | | | |
| --- | --- | --- | --- | --- | --- | --- | --- | --- |
|  | | Household spending (<median) | |  | | Household spending (<median) | | |
| NPNLW | | Mean | SD |  | | Mean | | SD |
| Intake of total zinc (mg/d) | | 3.8 | 2.2 |  | | 4.74^**^ | | 2.2 |
| Intake of animal zinc (mg/d) | | 1.03 | 0.8 |  | | 1.59^**^ | | 1.1 |
| Regression finding: Haemoglobin was positively associated with serum zinc in women | | | | | | | Zinc deficiency (%) | |
| Anaemic^†^ (%) |  | | | | 69^*^ | | | |
| Non-anaemic(%) |  | |  | 54 | | | | |

^†^ Anaemia is defined as haemoglobin<12 gm/dl

^*^p=0.02; ^**^p=0.001

Supplementary Table 2: comparative intake of zinc, phytate and P-Z molar ratio between two contemporary studies of Bangladesh

|  | NMS 2011-12^(1)^ | | Arsenault et al 2010^(2)^ | |
| --- | --- | --- | --- | --- |
|  | PSAC (2-<5 y) | NPNLW (15-49) | Children 2-4 y | Female caregiver |
| Zinc(mg/d) | 3.1 | 4.3 | 2.5 | 5.5 |
| Phytate(mg/d) | 234.1 | 498.1 | 260 | 586 |
| P-Z molar ratio | 9.0 | 12.0 | 10.6 | 10.8 |

Supplementary Table 3: Sample size for estimation of zinc deficiency in the national micronutrient survey

| Population | p | z | d | Design effect | Adjustment for non response | % in population | Sample size in 1 stratum | Sample size in 3 strata | Adjusted sample size in 3 strata |
| --- | --- | --- | --- | --- | --- | --- | --- | --- | --- |
| PSAC | 0.24^†^ | 1.96 | 0.07 | 2 | 1.2 | 0.1 | 323 | 969 | 1050 |
| NPNLW | 0.73 | 1.96 | 0.06 | 2 | 1.2 | 0.25 | 505 | 1515 | 1500 |

^†^Based on prevalence of low serum zinc observed in a contemporary study ^(2)^

Reference

1. National Micronutrient Survey 2011-12, Final Report. Dhaka, Bangladesh: Institute of Public Health Nutrition, United Nation Children’s Fund (UNICEF), icddr,b and Global Alliance for Improved Nutrition(GAIN)
2. Arsenault JE, Yakes EA, Hossain MB et al (2010). The Current High Prevalence of Dietary Zinc Inadequacy among Children and Women in Rural Bangladesh Could Be Substantially Ameliorated by Zinc Biofortification of Rice; *J. Nutr.* **140:** 1683–1690
